# Randomised Comparison of the AMBU AuraOnce Laryngeal Mask and the LMA Unique Laryngeal Mask Airway in Spontaneously Breathing Adults

**DOI:** 10.1155/2012/405812

**Published:** 2012-02-29

**Authors:** Daryl Lindsay Williams, James M. Zeng, Karl D. Alexander, David T. Andrews

**Affiliations:** Department of Anaesthesia and Pain Management, Royal Melbourne Hospital, Parkville 3050, Australia

## Abstract

We conducted a randomised single-blind controlled trial comparing the LMA-Unique (LMAU) and the AMBU AuraOnce (AMBU) disposable laryngeal mask in spontaneously breathing adult patients undergoing general anaesthesia. Eighty-two adult patients (ASA status I–IV) were randomly allocated to receive the LMAU or AMBU and were blinded to device selection. Patients received a standardized anesthetic and all airway devices were inserted by trained anaesthetists. Size selection was guided by manufacturer recommendations. All data were collected by a single, unblinded observer. When compared with the LMAU, the AMBU produced significantly higher airway sealing pressures (AMBU 20 ± 6; LMAU 15 ± 7 cm H_2_O; *P* = 0.001). There was no statistical difference between the two devices for overall success rate, insertion time, number of adjustments, laryngeal alignment, blood-staining, and sore throat (*P* ≥ 0.05). The AMBU AuraOnce disposable laryngeal mask provided a higher oropharyngeal leak pressure compared to the LMA Unique in spontaneously breathing adult patients.

## 1. Introduction

In 1988, Dr. Archie Brain introduced a reusable supraglottic airway device, the Laryngeal Mask Airway Classic (LMAC), a proven safe and effective device in airway management. There are several single-use disposable alternatives to the LMAC. They differ in shape, stiffness, and cuff properties to the LMAC [1]. We conducted a randomized comparison of two disposable supraglottic airways and their seal efficacy: the Laryngeal Mask Airway-Unique (LMAU; Laryngeal Mask Company, Henley-on-Thames, United Kingdom) and the AMBU AuraOnce Disposable Laryngeal Mask (AMBU; AMBU A/S, Denmark). The LMAU is made from a medical grade PVC compound and is similar in design to the LMAC. The AMBU is constructed from a single piece PVC mould and is available in a full range of paediatric and adult sizes. It incorporates a 90-degree preformed curvature designed to better approximate airway anatomy [2] and lacks the aperture bars of the LMAU.

## 2. Materials and Methods

After receiving institutional review board approval (Royal Melbourne Hospital), written informed consent was obtained during the preanaesthetic assessment on 82 consecutive patients aged above 18 years (ASA I–IV) [3] undergoing spontaneous ventilating general anaesthesia. Patients undergoing peripheral surgery in orthopaedics and plastic surgery and patients undergoing breast (general) or urological surgery were deemed suitable for recruitment. Patients were excluded if they had contraindications to the use of a supraglottic airway (BMI > 40, interincisor distance <2.5 cm, or aspiration risk) or had a contraindication to our anaesthetic protocol. Patients were allocated to either the LMAU or AMBU groups by a pregenerated random number sequence concealed in a sealed opaque envelope. This was opened after informed consent and patients remained blinded to their group allocation. Weight-based sizing of the airway devices was used based on manufacturer recommendations (size 3: 30–50 kg; size 4: 50–70 kg; size 5 > 70 kg). All participating consultant anaesthetists had >200 previous clinical attempts with an LMAU equivalent device (LMAC). To ensure adequate familiarity with the AMBU device, all anaesthetists completed an AMBU education program consisting of interactive tutorial and successful completion of 10 insertions with the AMBU on a manufacturer recommended part-task airway trainer. Routine preinsertion tests were performed according to the manufacturers' protocols [2, 4]. No premedication was given. Patients were preoxygenated and anaesthesia was induced with propofol (1-2 mg/kg), fentanyl (1–3 *μ*g/kg), ± midazolam (0.025–0.05 mg/kg) at the discretion of the anaesthetist. Routine anaesthetic monitoring was instituted as per Australian and New Zealand College of Anaesthetists guidelines for general anaesthesia [5].

Following induction of anaesthesia patients underwent assisted bag-mask ventilation with 100% oxygen and sevoflurane. After loss of lash reflex and loss of jaw tone, the airway device was inserted and fixed according to manufacturer instruction [2, 4]. The cuff was filled with air to the maximum manufacturers recommended volume (size 3, 20 mls; size 4, 30 mls; size 5, 40 mls), and the intracuff pressure was measured using a three-way stopcock and calibrated aneroid manometer. Three attempts at insertion were allowed. During spontaneous ventilation, anaesthesia was maintained using sevoflurane (1–3%) and an oxygen-air mixture to achieve an inspired oxygen concentration of 70–80%. Metaraminol was administered for any systemic arterial hypotension. Analgesia was provided by titrated boluses of fentanyl. Following conclusion of surgery, the anaesthetic gases were replaced by 100% oxygen and the patient transferred to recovery room. The device was removed with the return of airway reflexes.

All data were collected by a single, unblinded, independent observer. The patient's age, sex, height and weight, American Society of Anesthesiologists-Physical Status (ASA) [3], device size and type, and duration of surgery were recorded. An effective airway was defined by resistance to further downward motion, chest wall movement, presence of end tidal side-stream CO_2_ waveform, and movement of the reservoir bag during spontaneous ventilation. A failed insertion attempt was defined as complete withdrawal from the mouth following an unsuccessful placement. The number of insertion attempts was recorded, and after three attempts, the device was regarded as failed and a cLMA was then inserted without further data collection. The total time of insertion was defined by the moment the device was picked up to the first end tidal side-stream CO_2_ trace.

Our primary endpoint of oropharyngeal leak pressure (OLP) was determined using the manometric stability test with a fresh gas flow of 3 L/min against a closed pressure-limiting valve of the anaesthetic circuit. The airway pressure (maximum of 30 cm H_2_O) was recorded when equilibrium was obtained. After insertion, laryngeal alignment was assessed with fibreoptic bronchoscope (Olympus LF-GP, Olympus Optical Company, Japan) inserted to the aperture of the device via a self-sealing diaphragm. The fibreoptic view was graded according to an established scoring system for direct laryngoscopy [6]. Respiratory complications such as desaturations (pulse oximetry <95%), hiccups or stridor were noted. Cuff pressure was recorded at 5-minute intervals. The device was inspected for blood on removal and patients were directly questioned for sore throat using a dichotomous rating (present or absent) on discharge from the recovery room.

Sample size was obtained by performing a prospective power analysis with oropharyngeal leak pressure as the primary endpoint. The sample size was calculated to project a change of 20% in the primary outcome variable based on a previous peer-reviewed trial result for the LMAU of 25 ± 6 mmHg (mean ± S.D) [7], with a type I error 0.05 and a power of 95% to reject the null hypothesis. All quantitative data were found to be parametric and were examined by two-sided Student's *t*-tests for single comparisons and ANOVA for multiple comparisons. Cuff pressure changes were analysed by repeated measures ANOVA (SYSTAT v.7, SPSS Inc. Chicago). Qualitative data were assessed by Chi-square or Fisher exact test where appropriate. All data are presented as mean standard deviation (range) or numbers (%). *P* < 0.05 was considered statistically significant.

## 3. Results

Ninety-nine patients were identified as eligible for the study, eight patients declined consent, two patients had their surgery postponed, and seven patients were excluded by the treating anaesthetist, leaving eighty-two consecutive patients who were consented for the study. In three patients, equipment (fibreoptic bronchoscope) was unavailable, resulting in seventy-nine patients being randomised. At baseline, groups were similar (Table 1). The primary endpoint of oropharyngeal leak pressure (OLP) was completed in all patients except one in the AMBU group, where an airway could not be established after three insertion attempts. This was recorded as a failed airway in the AMBU group; however postoperative data were analysed on an intention-to-treat basis. The OLP for the AMBU (20 ± 6 cm H_2_O) was higher than for the LMAU (15 ± 7 cm H_2_O; *P* = 0.001) (Table 2). Of the secondary endpoints, a successful airway was established in all patients of the LMAU group. One patient had a failed airway in the AMBU group. Time to insertion, successful insertions, laryngeal alignment, blood staining, and sore throat were similar between groups (Table 2). Cuff pressures were similar for both devices (LMAU 84 ± 39 mmHg; AMBU 98 ± 36 mmHg; *P* = 0.473). Cuff pressure in the LMAU changed slightly over time whilst the AMBU remained stable (LMAU −7 ± 8 mmHg, AMBU 0 ± 3 mmHg, *P* = 0.01). Respiratory complications such as hiccups (LMAU *n* = 1, AMBU *n* = 1; *P* = 0.766), stridor (LMAU *n* = 3, AMBU *n* = 4; *P* = 0.529), and oxygen desaturations below 95% (LMAU *n* = 0, AMBU *n* = 1; *P* = 0.513) were similar between groups.

## 4. Discussion

Our study demonstrated higher oropharyngeal leak pressures in the AMBU compared to the LMAU. This may be due to the 90-degree angulation of the tube as described by Vaida [8] and is consistent with other recently published results of Shariffuddin and Wang [10] and Francksen et al. [9]. The mean oropharyngeal leakage pressure achieved in the AMBU group (20 ± 6 cm H_2_O) was similar to that of Shariffuddin (19 ± 7.5 cm H_2_O) but was lower than that of Lopez, Francksen, Gernoth and Hagberg (32.2 ± 6.8; 21 (13–40); 25.6 ± 5.2;  24 ± 5.5 cm H_2_O) [9, 11–13]. Our LMAU sealing pressure was also lower than that of other studies [7, 11, 14, 15]. These differences may be accounted for by a variety of mechanisms. We only allowed a maximum airway pressure of 30 cm H_2_O when performing the manometric stability test. Also, the injection of a maximum volume into the cuff (and subsequent higher cuff pressures) may have increased oropharyngeal leak due to cuff overinflation [14, 16]. Size selection may also play a role in oropharyngeal leakage pressure [17–19]. We use weight-based sizing as per manufacturer's recommendations, which resulted in 23 patients (29%) receiving size 3 masks, which is known to be associated with lower OLPs [17–19]. The differences may also be due to our large cohort of anaesthetists rather than a single experienced anaesthetist for insertion of the device [14]. 

Both devices performed similarly in establishing an adequate airway with first insertion (success rates AMBU 85%, LMAU 82%) and this is similar to other recent studies [10, 11, 13, 20]. There was a single failure within the AMBU group when there was easy insertion but inability to ventilate [10, 20]. Ng and Shariffuddin have hypothesized that these failures may be due to epiglottic downfolding on insertion of the AMBU [10, 20]. This is further supported effective ventilation following insertion with an LMAC, which has epiglottic aperture bars to prevent such an occurrence. This failure may be of consequence in the emergency setting where early establishment of ventilation is critical and other devices are not available (this aspect was not examined for directly in our study).

It has been previously suggested that the AMBU may be easier to insert in neck-immobilised patients [12], and the LMAUs PVC material may be more adherent to airway mucosa [21]. These factors may allow easier insertion in the AMBU. We found a trend toward faster insertion in the AMBU (LMAU 32 ± 16 s; AMBU 27 ± 10 s; *P* = 0.111) but this was not statistically significant, however our study was not powered to detect this, and further large-scale studies are needed.

The rate of vocal cord visualisation was similar in both groups (LMAU 79%; AMBU 78%; *P* = 0.613). This result is comparable to previous AMBU [10, 13] and LMAU [15, 22] studies.

Good anatomical alignment has been suggested to improve airway patency [23, 24] but other studies have shown no effect on device function [25]. The absence of aperture bars does not seem to alter the laryngeal alignment; however the absence of bars may aid insertion of instruments in the airway tube [13] such as assisted endotracheal intubation [26].

Both devices maintained stable cuff pressures. This finding is expected, given that we did not use N_2_O, and is similar to the results of Maino et al. [27]. Although our initial cuff pressures were higher (LMAU 84 ± 39 mmHg; AMBU 98 ± 36 mmHg; *P* = 0.473) than other studies [7, 10, 13–15, 22, 25, 28–31] due to injection of a maximal volume of air, we did not observe a greater incidence of airway trauma or sore throat. There is considerable debate in the literature about whether higher intracuff pressures actually equate to higher airway mucosal pressures and higher incidences of postoperative sore throat [32–36].

Our study has several limitations. The anaesthetists at our institution were significantly more experienced with LMAC and the LMAU compared with the AMBU. We attempted to minimise this effect by instituting a targeted education program; however there still may have been a learning curve with the AMBU. Second, our anaesthetists could not be blinded to the airway intervention. Bias was minimised by using a standard insertion technique for each device and an independent observer to collect data. Finally, our study was conducted only on adult patients in the operative setting and is not applicable to paediatric patients or emergency airway scenarios.

## 5. Conclusion

Our study found the AMBU to provide higher oropharyngeal leakage pressure than the LMAU in spontaneously breathing patients undergoing general anaesthesia. Overall success rates, insertion times, laryngeal alignment, and postoperative sore throat were statistically similar between both devices.

## Figures and Tables

**Table 1:** Patient demographics, surgery type, and airway sizing.

	LMAU (*n* = 38)	AMBU (*n* = 41)	*P*-value
Age (Years)	4620 (19–80)	4516 (19–80)	0.707
Weight (kg)	7213	7516	0.421
Height (cm)	17211	16812	0.114
BMI (kg/m^2^)	244	265	0.054
Male Gender	19 (50)	18 (44)	0.746
ASA (I/II/III/IV)	11/22/5/0	16/20/4/1	0.635
Surgery Type			0.875
General	14 (37)	21 (51)	
Plastics	12 (32)	13 (32)	
Orthopaedics	8 (21)	5 (12)	
Urology	4 (10)	2 (5)	
Airway Size (3/4/5)	14/19/5	19/16/6	0.738
Duration Surgery (mins)	3722 (15–110)	4620 (10–130)	0.375

All data are presented as mean standard deviation (range) or numbers (%). LMAU: LMA Unique laryngeal mask, AMBU: AMBU AuraOnce laryngeal mask, BMI: body mass index, ASA: American Society of Anesthesiologists physical status score.

**Table 2:** Results.

	LMAU (*n* = 38)	AMBU (*n* = 41)	*P*-value
Insertion success			
First time	31 (82)	35 (85)	0.738
Overall	38 (100)	40 (98)	0.519
Insertion time (s)	3216 (11–95)	2710 (13–51)	0.111
OLP (cm H_2_O)	157 (4–30)	206 (5–30)	0.001
Laryngeal Alignment	*n* = 38	*n* = 40	
I	21 (55)	21 (54)	
II	9 (24)	9 (23)	
III	7 (18)	7 (18)	
IV	1 (3)	2 (5)	0.613
Blood Staining	3 (8)	4 (10)	0.543
Sore Throat PACU	4 (11)	3 (8)	0.457

All data are presented as mean + standard deviation (range) or numbers (%). LMAU: LMA Unique laryngeal mask, AMBU: AMBU AuraOnce laryngeal mask, OLP: oropharyngeal leakage pressure, laryngeal alignment (I: visualise entire vocal cords, II: posterior component of cords visible, III: epiglottis but no cords visible, IV: no vocal cords or epiglottis visible).
